# A Hypothetical PM_2.5_ Intervention for the Risk of Hospitalization for Cardiovascular Diseases

**DOI:** 10.1001/jamanetworkopen.2025.39862

**Published:** 2025-10-28

**Authors:** Chengyi Lin, Lingzhi Chu, Riyang Liu, Antonio Gasparrini, Andrew T. DeWan, Laura Forastiere, Kai Chen

**Affiliations:** 1 Department of Environmental Health Sciences, Yale School of Public Health, New Haven, Connecticut; 2 Yale Center on Climate Change and Health, Yale School of Public Health, New Haven, Connecticut; 3 State Key Laboratory of Pollution Control and Resource Reuse, School of the Environment, Nanjing University, Nanjing, China; 4 Environment & Health Modelling Laboratory, Department of Public Health Environments and Society, London School of Hygiene & Tropical Medicine, London, United Kingdom; 5 Yale Center for Perinatal, Pediatric and Environmental Epidemiology, Yale School of Public Health, New Haven, Connecticut; 6 Department of Chronic Disease Epidemiology, Yale School of Public Health, New Haven, Connecticut; 7 Department of Biostatistics, Yale School of Public Health, New Haven, Connecticut

## Abstract

**Importance:**

There is limited direct evidence of the effects of policies regarding ambient particulate matter with an aerodynamic diameter of 2.5 µm or less (PM_2.5_) on the risk of hospitalization for cardiovascular diseases (CVD). This evidence is essential for estimating the benefits of meeting specific PM_2.5_ standards in the regulatory impact analysis.

**Objective:**

To estimate the association of strengthening ambient PM_2.5_ standards with the risk of hospitalization for major CVD outcomes.

**Design, Setting, and Participants:**

This population-based study used data from the UK Biobank cohort and followed up the participants from January 1, 2015, to December 31, 2019. All participants were 60 years or older and had no history of hospitalization with a primary diagnosis of a specific CVD at baseline. Data were analyzed from August 1, 2022, to August 25, 2025.

**Exposures:**

Annual mean PM_2.5_ exposure was assigned based on a 1 × 1-km^2^ resolution PM_2.5_ model linked to participants’ residential locations.

**Main Outcomes and Measures:**

The main outcomes were the first hospitalization with a primary diagnosis of stroke, myocardial infarction, heart failure, or arrhythmia. Longitudinal targeted maximum likelihood estimation was used to estimate 5-year hospitalization risks under hypothetical PM_2.5_ interventions.

**Results:**

Among the 502 133 UK Biobank participants recruited from 2006 to 2010 (273 158 [54.4%] female), 307 202 participants met the eligibility criteria for stroke, 304 212 for myocardial infarction, 310 100 for heart failure, and 302 255 for arrhythmia. The median age was 68.0 (IQR, 64.6-71.5) years for stroke, myocardial infarction, and arrhythmia, 68.0 (IQR, 64.7-71.5) years for heart failure, with female participants ranging from 54.4% to 55.0% across cohorts. Compared with no intervention on PM_2.5_, implementing a stricter ambient PM_2.5_ standard would reduce the absolute risk of hospitalization for major CVD. It was estimated that for the hypothetical PM_2.5_ intervention of reducing PM_2.5_ exposure by 5% if it is above the threshold of 9 µg/m^3^, the estimated 5-year risk difference of hospitalization for stroke was −2.26 per mille (95% CI, −8.97 to −20.64 per mille); for myocardial infarction, −8.64 per mille (95% CI, −9.16 to −6.38 per mille); for heart failure, −3.20 per mille (95% CI, −4.16 to −1.25 per mille); and for arrythmia, −4.16 per mille (95% CI, −12.70 to 12.93 per mille). For the hypothetical PM_2.5_ intervention of reducing PM_2.5_ exposure by 5% if it is above the threshold of 12 µg/m^3^, the estimated 5-year risk difference of hospitalization for stroke was −1.54 per mille (95% CI, −2.21 to 0.73 per mille). However, the reduction in risk for arrhythmia was not statistically significant (−2.06 per mille [95% CI, −4.79 to 3.12 per mille]).

**Conclusions and Relevance:**

In this cohort study using data from the UK Biobank, the absolute risk reduction of hospitalization for stroke, myocardial infarction, heart failure, and arrhythmia due to hypothetical ambient PM_2.5_ interventions was quantified. The findings suggest the beneficial cardiovascular health impacts of further strengthening the current PM_2.5_ regulations in the United Kingdom.

## Key Points

**Question:**

Is implementation of policies with different exposure standards for ambient particulate matter with an aerodynamic diameter of 2.5 µm or less (PM_2.5_) associated with a risk of hospitalization for major cardiovascular diseases?

**Findings:**

In this cohort study of 502 133 participants with data from the UK Biobank, implementing a hypothetical intervention for reducing PM_2.5_ exposure by 5% if it is above the thresholds of 12 and 9 µg/m^3^ would significantly reduce the 5-year risk of hospitalization for stroke, heart failure, and myocardial infarction, but not for arrhythmia.

**Meaning:**

These findings suggest that further strengthening the current ambient PM_2.5_ regulations in the United Kingdom could provide benefits for cardiovascular health.

## Teaser

This cohort study evaluates the association of hypothetical ambient PM_2.5_ interventions with the risk of cause-specific hospitalization for 4 cardiovascular diseases in the large-scale population-based UK Biobank cohort.

## Introduction

The average human life expectancy has been lengthened in previous decades and is projected to increase in the future,^1^ but the healthy life expectancy at 60 years of age is not increasing as fast as the life expectancy at 60 years of age. Globally, from 2000 to 2019, life expectancy at 60 years of age has increased by 2.1 years, while healthy life expectancy at 60 years of age has only increased by 1.5 years.^2^ Among the causes of reduced quality of life for this population, cardiovascular diseases (CVD) play the largest role.^3^

It is well known that exposure to ambient particulate matter with an aerodynamic diameter of 2.5 µm or less (PM_2.5_) is a potential modifiable environmental risk factor for CVD,^4,5^ likely through mechanisms of vascular dysfunction, thrombosis, endothelial damage, tissue inflammation, plaque instability, and epigenomic changes, as found in toxicologic and epidemiologic studies.^6^ However, causal evidence on evaluating the effectiveness of interventions that modify PM_2.5_ levels on the risk of CVD has been limited, which may contribute to the limited attention given to ambient PM_2.5_ in the CVD prevention guidelines.^7^

To address the causal question on the effectiveness of an intervention, a randomized clinical trial is often preferred. However, it is impractical and unethical to conduct a randomized clinical trial to evaluate the effectiveness of different ambient PM_2.5_ standards in a large population under clinical conditions. Instead, several causal inference frameworks have been proposed to leverage observational data for quantifying effects of interventions. These causal inference frameworks share the following components: (1) State the causal question, (2) Specify the causal quantity that could answer the causal question, (3) Specify the causal assumptions and the study design that makes the causal assumption more plausible, (4) Translate the causal quantity to a statistical quantity that can be estimated using the observational data under the causal assumptions, and (5) Evaluate the tenability of the causal interpretation.^8^

Traditionally, the Cox proportional hazards model has been used extensively in prior research^4,5^ to investigate the association between ambient PM_2.5_ exposure and the risk of CVD. However, the traditional Cox proportional hazards–based analysis, due to its nature of quantifying associations, is not designed to address the causal question concerning the potential effect of a hypothetical PM_2.5_ intervention on the risk of CVD.^9^ Moreover, given that the time-varying confounders may be affected by previous exposure (ie, the treatment-confounder feedback) in longitudinal studies, the time-dependent Cox proportional hazards model will produce biased estimates when the treatment-confounder feedback exists and the time-varying confounders adjusted for are posttreatment variables.^10^ To handle time-varying confounders that could be posttreatment variables, the longitudinal targeted maximum likelihood estimation (LTMLE) was proposed.^11^ LTMLE offers several other advantages, including its doubly robust property and the capability of incorporating machine learning algorithms into the estimation.^12^

To provide more direct evidence to support the decision-making process regarding whether to tighten the ambient PM_2.5_ standard by following the causal inference framework using observational data,^8^ we herein evaluated the association of hypothetical ambient PM_2.5_ interventions with the risk of cause-specific hospitalization for 4 CVD outcomes (ie, stroke, myocardial infarction, heart failure, and arrhythmia) using the large-scale population-based UK Biobank cohort. We applied the LTMLE method in comparing the counterfactual 5-year risk of a CVD outcome if the study population had experienced a hypothetical PM_2.5_ intervention with the 5-year risk under the observed PM_2.5_ exposure.

## Methods

### Study Population

The UK Biobank is a prospective cohort with 502 133 participants aged 37 to 73 years at the time of recruitment between 2006 to 2010 through volunteer-based sampling in the United Kingdom.^13^ Race and ethnicity were self-reported as Asian, Black, Chinese, White, multiracial, or other race or ethnicity (no additional information available), which were collected to characterize the study population and evaluate the generalizability of the findings. The UK Biobank received ethical approval as a Research Tissue Bank from the North West Multi-Centre Research Ethics Committee, and all participants provided written informed consent. This cohort study followed the Strengthening the Reporting of Observational Studies in Epidemiology (STROBE) reporting guideline.

For each CVD subtype (ie, stroke, myocardial infarction, heart failure, or arrhythmia), participants 60 years or older without a preceding hospitalization including a primary diagnosis of the specific CVD subtype at baseline (year 2015) were eligible for the analysis. Participants without complete data on ambient PM_2.5_ exposure were excluded (eFigures 1-4 in Supplement 1). The final cohorts included 307 202 participants for stroke, 304 212 for myocardial infarction, 310 100 for heart failure, and 302 255 for arrhythmia. These participants were followed up from January 1, 2015, to the earliest occurrence of the specified outcome, death, loss to follow-up, or the end of follow-up on December 31, 2019.

### PM_2.5_ Exposure Estimation and Covariates

**Figure 1.**
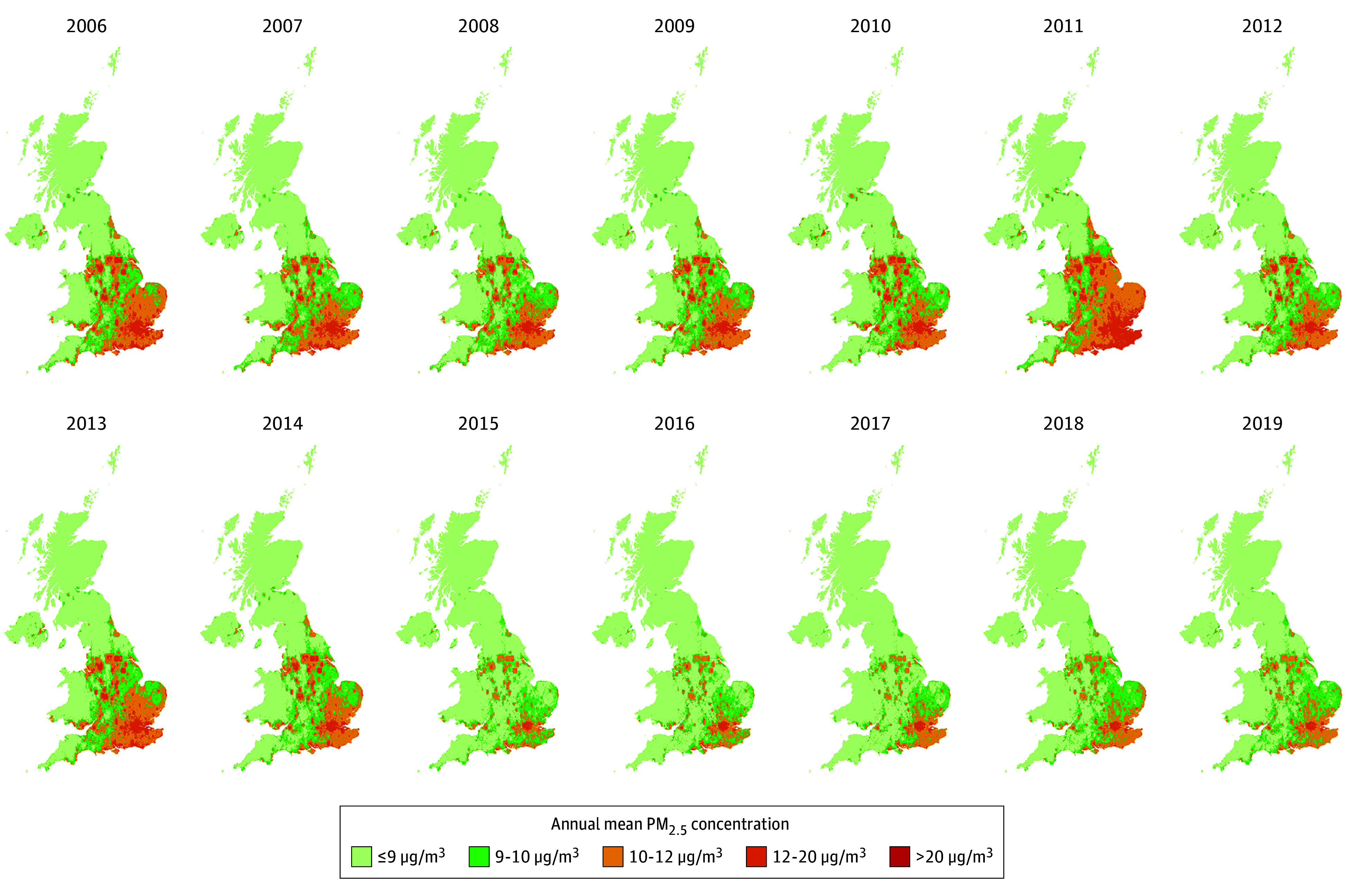
Modeled Annual Mean PM_2.5_ Concentration in the United Kingdom From 2006 to 2019 PM_2.5_ indicates ambient particulate matter with an aerodynamic diameter of 2.5 µm or less.

Individual-level ambient PM_2.5_ exposure from 2006 to 2019 (Figure 1) was derived using high-resolution (1 × 1 km^2^) monthly PM_2.5_ estimates developed in a previous study that included investigators from our group.^14^ The estimates were generated using a light gradient boosting machine that captured nonlinear and high-order associations between in situ PM_2.5_ observations and multisource geospatial datasets to estimate PM_2.5_ concentrations across the study domain. The model achieved a 10-fold by-year cross-validation coefficient of determination of 0.82 at the monthly level, demonstrating its suitability for health effect analysis (eAppendix 1 in Supplement 1). We used bilinear interpolation to calculate the individual-level monthly exposure using the nearest 4 grids for each residential location, with the address changes tracked throughout the follow-up period (eAppendix 2 in Supplement 1). The individual-level monthly mean temperature exposure was derived from the fifth-generation European Centre for Medium-Range Weather Forecasts atmospheric reanalysis of the global climate monthly mean data at a spatial resolution of 0.25° × 0.25° using the grid covering the residential location.^15^ The time-varying annual mean exposure was calculated as the 12-month mean of the monthly exposure values. The annual gross disposable household income per head index, an indicator of economic welfare, was provided by the Office for National Statistics at International Territorial Level 3 in the 1997 to 2021 edition of the regional gross disposable household income dataset.^16^ We calculated the annual population density at International Territorial Level 3 as the ratio of the midyear residential population, sourced from the Office for National Statistics, to the respective regional area.

In addition to the aforementioned time-varying covariates, we used time-invariant covariates collected by questionnaire conducted at the first visit to assessment centers, including sex (female or male), birth year and month, educational attainment (coded as with or without a college degree or unknown), mean household income before tax (coded as <£18 000, £18 000-£30 999£, £31 000-£51 999, £52 000-£100 000, >£100 000, do not know, or unknown), smoking status (coded as current smoker, former smoker, never smoker, or unknown), and self-reported family history of CVD (coded as yes or no). The categories of unknown include individuals who preferred not to answer or had missing data. Since body mass index (BMI) was not measured annually, we used the value recorded at the participant’s first visit to the assessment center. The modeled annual mean exposure to nitrogen dioxide and nitrogen oxides for the year 2010 is another time-invariant covariate available in the UK Biobank dataset. These exposure data were developed by the European Study of Cohorts for Air Pollution Effects with a land use regression model and linked to the residential addresses of each participant.^17^ We selected these covariates as confounders based on substantive knowledge from the literature regarding the association between long-term exposure to PM_2.5_ and CVD.^4,18^

### Outcome Definition

Using codes from the *International Classification of Diseases, Ninth Revision* (*ICD-9*), and *International and Statistical Classification of Diseases, Tenth Revision* (*ICD-10*), we identified the earliest date of hospitalization with a primary diagnosis for stroke (*ICD-9* codes 430, 431, 434, and 436; *ICD-10* codes I60, I61, I63, and I64), myocardial infarction (*ICD-9* codes 410, 411.0, 412, and 429.7; *ICD-10* codes I21-I23, I24.1, and I25.2), heart failure (*ICD-9* code 428; *ICD-10* code I50), or arrhythmia (*ICD-9* code 427; *ICD-10* codes I47-I49) from the hospital inpatient data. We identified the date of death from death registries for individuals who died after enrollment.

### Statistical Analysis

We aim to answer the question of what the associations of hypothetical PM_2.5_ interventions are with the risk of hospitalization with a primary diagnosis of major CVDs. The effect sizes are measured by comparing the difference in the cause-specific cumulative incidence (risk) of a CVD outcome under a hypothetical PM_2.5_ intervention compared with no invention on PM_2.5_ (ie, ambient PM_2.5_ exposure in the natural course), with death as the competing event.^19^ Under the hypothetical PM_2.5_ interventions with different annual mean PM_2.5_ concentration thresholds, the individual-level exposures to ambient PM_2.5_ are reduced by 5% if they surpass it during a follow-up year; otherwise, the PM_2.5_ exposures are not under intervention.^20,21^ The 3 thresholds values assessed were 12 µg/m^3^ according to the England’s 2028 interim target, 10 µg/m^3^ according to England’s 2040 and Scotland’s 2020 targets, and 9 µg/m^3^ according to the primary annual standard by the US Environmental Protection Agency.^22,23^

These risks of CVD outcomes under hypothetical PM_2.5_ interventions can be estimated under the assumptions of no unmeasured confounding, positivity, consistency, and interference.^24^ We used the LTMLE to estimate the risks of CVD outcomes under different hypothetical PM_2.5_ interventions (eMethods in Supplement 1).^24,25,26^ We used the cross-fitted version of LTMLE with 5 folds.^27,28^ We conducted subgroup analyses by sex and age at the baseline year 2015 (≥68 or <68 years). To assess the robustness of the results to residual confounding, we performed further analyses by additionally adjusting for the annual mean exposure to nitrogen dioxide and nitrogen oxides for the year 2010, family history of CVD, and BMI.

We used the lmtp package to implement the LTMLE in R, version 4.3.2 (R Program for Statistical Computing).^29^ For the estimation of nuisance parameters, we used AutoGluon, version 1.4.0 (Amazon Science Team). Data were analyzed from August 1, 2022, to August 25, 2025. Two-sided *P* < .05 was considered statistically significant.

## Results

Among the 502 133 participants recruited in the UK Biobank from 2006 to 2010 (273 158 [54.4%] female and 228 975 [45.6%] male), 307 202 participants met the eligibility criteria for stroke, 304 212 met the eligibility criteria for myocardial infarction, 310 100 met the eligibility criteria for heart failure, and 302 255 met the eligibility criteria for arrhythmia (eFigures 1-4 in Supplement 1). During the 5-year follow-up from 2015 to 2019, 3785 participants had stroke, 3490 had myocardial infarction, 2015 had heart failure, and 5700 had arrhythmia.

Table 1 shows the characteristics of all eligible participants in the baseline year 2015. The median (IQR) age was 68.0 (IQR, 64.6-71.5) years for stroke, myocardial infarction, and arrhythmia and 68.0 (IQR, 64.7-71.5) years for heart failure, with female participants ranging from 54.4% to 55.0% across cohorts. For the outcome of stroke, 88 937 participants (29.0%) had a college degree; for myocardial infarction, 88 385 (29.1%) had a college degree; for heart failure, 89 607 (28.9%) had a college degree; and for arrhythmia, 87 353 (28.9%) had a college degree. The annual PM_2.5_ concentration in the baseline year 2015 was 10.39 (IQR, 9.41-11.36) µg/m^3^ for the cohort with the outcome of stroke, heart failure, or arrhythmia. For the cohort with myocardial infarction as the outcome, the annual PM_2.5_ concentration in the baseline year 2015 was 10.40 (IQR, 9.41-11.37) µg/m^3^.

**Table 1.** Baseline Characteristics of Eligible Participants in the UK Biobank Cohort

Characteristic	Outcome eligibility^a^			
	Stroke (n = 307 202)	Myocardial infarction (n = 304 212)	Heart failure (n = 310 100)	Arrhythmia (n = 302 255)
Age in 2015, median (IQR), y	68.0 (64.7-71.5)	68.0 (64.6-71.5)	68.0 (64.6-71.5)	68.0 (64.6-71.5)
Sex, No. (%)				
Female	167 344 (54.5)	167 438 (55.0)	168 612 (54.4)	165 544 (54.8)
Male	139 858 (45.5)	136 774 (45.0)	141 488 (45.6)	136 711 (45.2)
Educational attainment, No. (%)				
College degree	88 937 (29.0)	88 385 (29.1)	89 607 (28.9)	87 353 (28.9)
No college degree	211 876 (69.0)	209 486 (68.9)	214 020 (69.0)	208 626 (69.0)
Unknown^b^	6389 (2.1)	6341 (2.1)	6473 (2.1)	6276 (2.1)
Race and ethnicity, No. (%)				
Asian	8635 (2.8)	8571 (2.8)	8696 (2.8)	8508 (2.8)
Black	1150 (0.4)	1127 (0.4)	1158 (0.4)	1132 (0.4)
Chinese	648 (0.2)	647 (0.2)	652 (0.2)	643 (0.2)
White	283 552 (92.3)	280 768 (92.3)	286 257 (92.3)	278 953 (92.3)
Multiracial	9525 (3.1)	9446 (3.1)	9615 (3.1)	9373 (3.1)
Other^c^	1801 (0.6)	1778 (0.6)	1809 (0.6)	1789 (0.6)
No. Missing	1891	1875	1913	1857
Mean total household income before tax, No. (%)				
<£18 000	70 095 (22.8)	69 203 (22.7)	70 984 (22.9)	69 053 (22.8)
£18 000-£30 999	74 618 (24.3)	73 848 (24.3)	75 357 (24.3)	73 363 (24.3)
£31 000-£51 999	60 549 (19.7)	59 972 (19.7)	61 017 (19.7)	59 522 (19.6)
£52 000-£100 000	38 556 (12.6)	38 274 (12.6)	38 797 (12.5)	37 942 (12.6)
>£100 000	9435 (3.1)	9366 (3.1)	9478 (3.1)	9249 (3.1)
Do not know	14 849 (4.8)	14 814 (4.9)	15 028 (4.8)	14 663 (4.9)
Unknown^b^	39 100 (12.7)	38 735 (12.7)	39 439 (12.7)	38 463 (12.7)
Smoking status, No. (%)				
Never	159 417 (51.9)	158 646 (52.1)	160 661 (51.8)	157 028 (52.0)
Previous	119 613 (38.9)	117 845 (38.7)	120 787 (39.0)	117 148 (38.8)
Current	26 351 (8.6)	25 912 (8.5)	26 807 (8.6)	26 295 (8.7)
Unknown^b^	1821 (0.6)	1809 (0.6)	1845 (0.6)	1784 (0.6)
Family history of CVD, No. (%)	187 331 (61.0)	191 560 (63.0)	191 560 (61.8)	189 734 (62.8)
BMI, mean (IQR)	27.5 (24.4-30.0)	27.5 (24.4-30.0)	27.5 (24.4-30.0)	27.5 (24.4-30.0)
No. missing	6165	6108	6195	5565
Modeled annual mean exposure to nitrogen dioxide for 2010, mean (IQR), µg/m^3^	26.24 (21.13-30.66)	26.25 (21.13-30.67)	26.24 (21.13-30.66)	26.24 (21.13-30.67)
No. missing	3696	3659	3728	3646
Modeled annual mean exposure to nitrogen oxides for 2010, mean (IQR), µg/m^3^	43.27 (33.76-49.80)	43.28 (3.76-49.82)	43.28 (33.77-49.82)	43.28 (33.77-49.82)
No. missing	3696	3659	3728	3646
Annual PM_2.5_ concentration for baseline 2015, mean (IQR), µg/m^3^	10.39 (9.41-11.36)	10.40 (9.41-11.37)	10.39 (9.41-11.36)	10.39 (9.41-11.36)
Annual mean temperature for baseline 2015, mean (IQR), °C	10.10 (9.49-10.77)	10.10 (9.49-10.77)	10.10 (9.49-10.77)	10.10 (9.49-10.77)
Annual GDHI per head index for baseline 2015, mean (IQR)	98 (80-109)	98 (80-109)	98 (80-109)	98 (80-109)
Annual population density for baseline 2015, mean (IQR), person/m^2^	0.19 (0.05-0.22)	0.19 (0.05-0.22)	0.19 (0.05-0.22)	0.19 (0.05-0.22)

Abbreviations: BMI, body mass index (calculated as the weight in kilograms divided by the height in meters squared); GDHI, gross disposable household income; PM_2.5_, particulate matter with an aerodynamic diameter less than or equal to 2.5 µm.

^a^ Percentages have been rounded and may not total 100.

^b^ Includes individuals who either selected “prefer not to answer” or had missing data.

^c^ Category was self-selected and includes no additional information.

Table 2 shows the estimated 5-year risk of hospitalization for major CVD outcomes under hypothetical ambient PM_2.5_ interventions among eligible participants in the UK Biobank from 2015 to 2019. For the threshold intervention of 12 µg/m^3^ compared with no intervention, the estimated 5-year risk difference of hospitalization for stroke was −1.54 per mille (95% CI, −2.21 to −0.73 per mille); for myocardial infarction, −1.41 per mille (95% CI, −1.98 to −0.67 per mille); for heart failure, −0.51 per mille (95% CI, −1.07 to 0.35 per mille); and for arrhythmia, −2.06 per mille (95% CI, −4.79 to 3.12 per mille). For the threshold intervention of 10 µg/m^3^ compared with no intervention, the estimated 5-year risk difference of hospitalization for stroke was −5.34 per mille (95% CI, −8.59 to 7.24 per mille); for myocardial infarction, −7.32 per mille (95% CI, −8.05 to −5.86 per mille); for heart failure, −2.15 per mille (95% CI, −3.55 to −0.35 per mille); and for arrhythmia, −9.05 per mille (95% CI, −12.59 to 1.28 per mille). For the threshold intervention of 9 µg/m^3^ compared with no intervention, the estimated 5-year risk difference of hospitalization for stroke was −2.26 per mille (95% CI, −8.97 to −20.64 per mille); for myocardial infarction, −8.64 per mille (95% CI, −9.16 to −6.38 per mille); for heart failure, −3.20 per mille (95% CI, −4.16 to −1.25 per mille) for heart failure; and for arrhythmia, −4.16 per mille (95% CI, −12.70 to 12.93 per mille).

**Table 2.** Estimated 5-Year Risk of Major Cardiovascular Diseases Under Hypothetical Interventions for Ambient Particulate Matter With an Aerodynamic Diameter Less Than or Equal to 2.5 µm Among Eligible Participants in the UK Biobank Cohort, 2015 to 2019

Hypothetical intervention	5-y Risk (95% CI), per mille	Risk difference (95% CI), per mille
**Stroke**		
No intervention	12.32 (11.92 to 12.72)	[Reference]
Threshold level, µg/m^3^		
≤12	10.78 (10.07 to 11.67)	−1.54 (−2.21 to −0.73)
≤10	6.98 (3.76 to 19.60)	−5.34 (−8.59 to 7.24)
≤9	10.06 (3.37 to 32.97)	−2.26 (−8.97 to 20.64)
**Myocardial infarction**		
No intervention	11.47 (11.08 to 11.84)	[Reference]
Threshold level, µg/m^3^		
≤12	10.07 (9.44 to 10.85)	−1.41 (−1.98 to −0.67)
≤10	4.15 (3.47 to 5.54)	−7.32 (−8.05 to −5.86)
≤9	2.83 (2.35 to 5.03)	−8.64 (−9.16 to −6.38)
**Heart failure**		
No intervention	6.50 (6.23 to 6.76)	[Reference]
Threshold level, µg/m^3^		
≤12	5.99 (5.37 to 6.91)	−0.51 (−1.07 to 0.35)
≤10	4.35 (2.95 to 6.89)	−2.15 (−3.55 to 0.35)
≤9	3.29 (2.40 to 5.20)	−3.20 (−4.16 to −1.25)
**Arrhythmia**		
No intervention	18.86 (18.36 to 19.35)	[Reference]
Threshold level, µg/m^3^		
≤12	16.80 (14.07 to 21.79)	−2.06 (−4.79 to 3.12)
≤10	9.81 (6.28 to 19.92)	−9.05 (−12.59 to 1.28)
≤9	14.70 (6.14 to 31.74)	−4.16 (−12.70 to 12.93)

**Figure 2.**
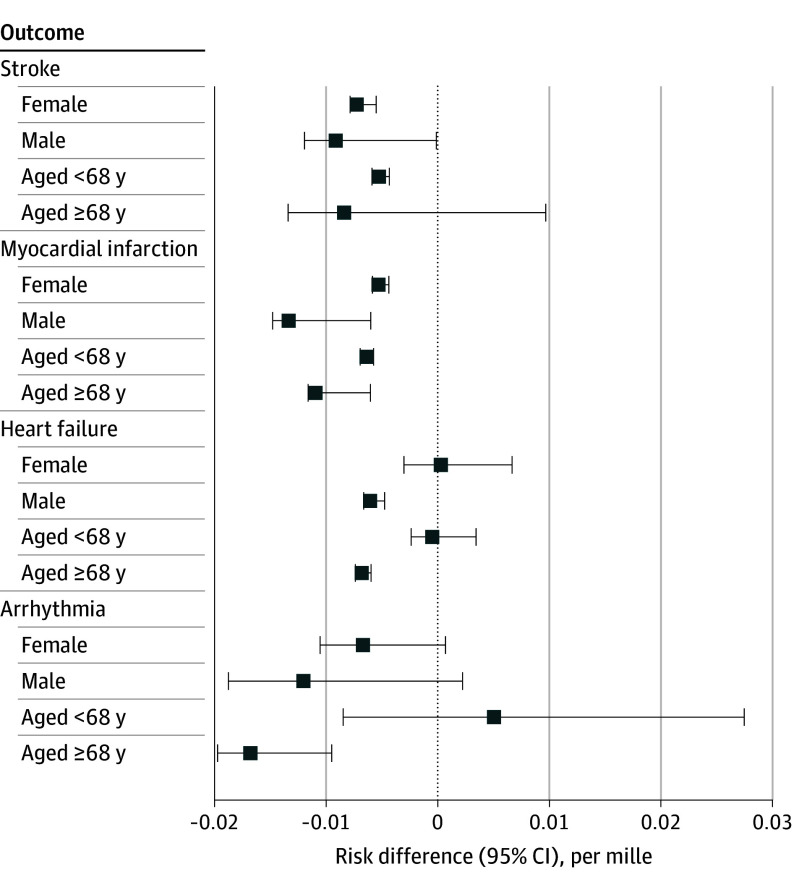
Estimated 5-Year Risk Difference of Hospitalization for Major Cardiovascular Diseases for the Hypothetical Intervention for Subgroups Participants included eligible participants in the UK Biobank cohort from 2015 to 2019. Subgroups include female and male sex and age younger than 68 years or 68 years and older. Intervention consists of reducing ambient particulate matter with an aerodynamic diameter of 2.5 µm or less (PM_2.5_) by 5% if it is above the threshold of 9 µg/m^3^. Numeric data are given in eTable 1 in Supplement 1.

**Figure 3.**
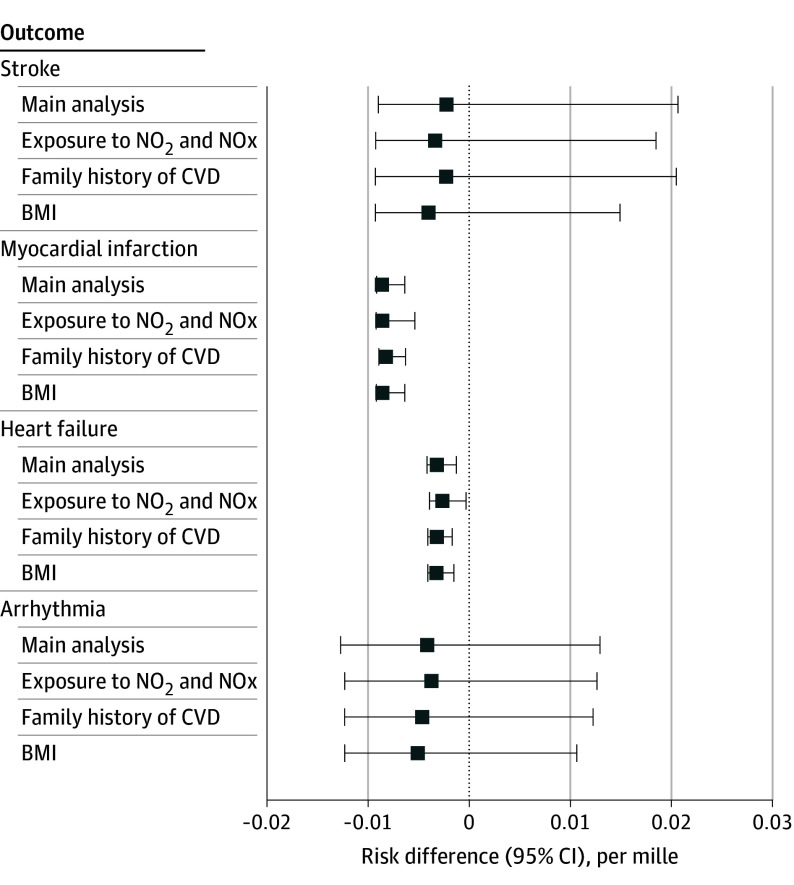
Estimated 5-Year Risk Difference of Hospitalization for Major Cardiovascular Diseases for the Main Analysis and Robustness Check Intervention consists of reducing ambient particulate matter with an aerodynamic diameter of 2.5 µm or less (PM_2.5_) by 5% if it is above the threshold of 9 µg/m^3^ compared with no PM_2.5_ intervention. Robustness checks include further adjustment for the modelled annual exposure to nitrogen dioxide (NO_2_) and nitrogen oxides (NOx) for the year 2010, family history of cardiovascular disease (CVD), and body mass index (BMI). Numeric data are given in eTable 2 in Supplement 1.

The estimated 5-year risk difference of hospitalization for the hypothetical intervention of reducing PM_2.5_ by 5% if it is above the 9-µg/m^3^ threshold compared with no intervention on ambient PM_2.5_ was larger among individuals who were 68 years or older at baseline compared with those younger than 68 years for myocardial infarction (−10.93 per mille [95% CI, −11.63 to −6.02 per mille] vs −6.39 per mille [95% CI, −6.96 to −5.75 per mille]) (*P* = .002), heart failure (−6.81 per mille [95% CI, −7.37 to −5.98 per mille] vs −0.47 per mille [95% CI, −2.38 to 3.42 per mille]) (*P* < .001), and arrhythmia (−16.78 per mille [95% CI, −19.71 to −9.49 per mille] vs 5.07 per mille [95% CI, −8.48 to 27.47 per mille]) (*P* = .002). The risk difference was larger among male compared with female participants for the outcomes of myocardial infarction (−13.36 per mille [95% CI, −14.79 to −6.01 per mille] vs −5.30 per mille [95% CI, −5.85 to −4.39 per mille]) (*P* < .001) and heart failure (−6.05 per mille [95% CI, −6.62 to −4.76 per mille] vs 0.27 per mille [95% CI, −3.01 to 6.67 per mille) (*P* = .002) (Figure 2 and eTable 1 in Supplement 1). The results of the robustness check, which included the further adjustment for the modeled annual mean exposure to nitrogen dioxide and nitrogen oxides for the year 2010, further adjustment for family history of CVD, and further adjustment for BMI, did not significantly differ from the main results (Figure 3 and eTable 2 in Supplement 1).

## Discussion

In this study, we used the UK Biobank cohort data to estimate the association of hypothetical ambient PM_2.5_ interventions with hospitalization for a primary diagnosis of major CVDs using the LTMLE. Our findings indicate a decreased 5-year risk of hospitalization with a primary diagnosis of stroke, myocardial infarction, or heart failure for the hypothetical intervention of reducing PM_2.5_ exposure by 5% if it is above the annual average standards of 12 µg/m^3^, 10 µg/m^3^, or 9 µg/m^3^ compared with no intervention on ambient PM_2.5_. However, the 5-year risk difference was not statistically significant for the hospitalization with a primary diagnosis of arrhythmia.

Although our study aims to answer casual questions regarding the effects of hypothetical PM_2.5_ interventions on CVD outcomes, our findings cannot be validated against an actual but unfeasible randomized clinical trial. However, our results are generally consistent with findings from corresponding observational studies.^4^ A recent study conducted on the same UK Biobank cohort using the Cox proportional hazards model^30^ found positive associations between long-term PM_2.5_ exposure and hospitalization for stroke, heart failure, and arrhythmia, but no association for myocardial infarction. A study conducted on a US national cohort of approximately 60 million participants^31^ found that the 3-year average PM_2.5_ exposure between 9 and 10 µg/m^3^ is associated with an increased risk of first hospitalization for ischemic heart disease, cerebrovascular disease, heart failure, and arrhythmia, compared with an exposure level less than 5 µg/m^3^. While our study found decreased 5-year risks of stroke, myocardial infarction, and heart failure for the hypothetical PM_2.5_ intervention, the risk difference was not statistically significant for the outcome of arrhythmia. This inconsistency may be attributable to the differences in the definition of the hypothetical PM_2.5_ intervention, the population characteristics, or the *ICD-9* and *ICD-10* codes used to classify the outcome. While generally consistent with previous findings regarding the associations between long-term exposure to ambient PM_2.5_ and the risks of CVD, our study provides a straightforward interpretation by explicitly defining the PM_2.5_ intervention and by using the risk difference (absolute risk reduction) as the effect measure.

### Strengths and Limitations

This study has several strengths. First, it provides a direct estimation of the effect of ambient PM_2.5_ regulations with different thresholds. Instead of estimating the exposure-response relationship between PM_2.5_ and CVD, we aimed to directly quantify the association with different limits for the ambient PM_2.5_ concentration on the risk of CVD hospitalization.^32^ Second, incorporated the risk difference (absolute risk reduction) of CVD hospitalization for PM_2.5_ interventions, a measure of public health impact that is more stable than the coefficient of PM_2.5_ obtained in the Cox proportional hazards models that do not apply inverse probability weighting for censoring.^33^ Third, we used a causal inference method (ie, LTMLE) to adjust for time-varying confounders, which helps avoid biased estimates that can arise from methods that stratify time-varying confounders when they are posttreatment variables (treatment-confounder feedback).^10^ In addition, we account for the competing event (ie, death) in a causal framework.^19,25^ Fifth, we leveraged a state-of-the-art AutoML framework, AutoGluon, for the estimation of nuisance parameters. In addition to its strong performance,^34^ AutoGluon efficiently handles large datasets in a reasonable time frame compared with the implementation of the SuperLearner package within the lmtp package, which makes it a promising tool for future research involving large-scale clinical data.

This study also has limitations. First, residual confounding may exist, although our results remained stable in the additional robustness check. We used ambient PM_2.5_ concentrations rather than personal exposure, as they were the primary exposure of interest and are less likely to be confounded by individual physiological and behavioral factors.^18^ Furthermore, the results are unlikely to change substantially after adjusting for neighborhood-level confounders, including material welfare, population density, and temperature. The robustness check showed that the estimates did not essentially change with further adjustment for modeled exposure to nitrogen dioxide and nitrogen oxides and BMI. Second, we set 2015 as the baseline year rather than earlier years and were unable to estimate the effects of hypothetical PM_2.5_ interventions with lower threshold values or those starting before 2015 due to the limited proportion of participants with low PM_2.5_ exposure levels, which may lead to violation of the positivity assumption. The limited overlap in the conditional probability densities of exposure under hypothetical intervention and no intervention will lead to inaccurate density ratio estimates in finite samples,^35^ thereby impeding the estimation of treatment mechanisms. Third, the residential address records collected in the UK Biobank contain some level of inaccuracy. Fourth, there may be outcome misclassification due to the use of electronic health records to identify outcomes. However, the accuracy of hospitalization primary diagnosis for stroke is relatively high, with a positive predictive value of 94% (95% CI, 88%-98%) in a validation study with a regional UK Biobank population of 17 249 participants.^36^ Fifth, we did not account for the chemical composition of PM_2.5_ in the definition of PM_2.5_ intervention. Given that some PM_2.5_ constituents were found to be associated with a greater risk of CVD hospitalization, the consistency assumption (ie, the treatment versions are well-defined) for causal inference may be violated.^37,38^ As a result, our effect estimates may differ from those observed under specific clinical interventions, such as stricter emission standards for vehicles and power plants. Sixth, we applied only the LTMLE estimator; future work may consider alternative estimators to assess robustness. Additionally, the generalizability of the estimates to the general UK population may be compromised due to differences in the eligible participants’ characteristics compared with the general population, such as a greater proportion of female participants, being healthier, and residing in less socioeconomically deprived areas.^39^

## Conclusions

In this cohort study, we used UK Biobank data to directly estimate the association of hypothetical PM_2.5_ interventions starting in late life with the risk of hospitalization for major CVD outcomes using LTMLE. Our results indicate that a more stringent ambient PM_2.5_ standard in the United Kingdom may lower the risk of hospitalization for stroke, myocardial infarction, and heart failure.

## Notes

Supplement 1. **eAppendix 1.** PM_2.5_ Estimates **eAppendix 2.** Historical Residential Location **eMethods.** Additional Detail on Statistical Analysis **eTable 1.** Estimated 5-Year Risk and Risk Difference of Hospitalization for Major Cardiovascular Diseases Between the Hypothetical Intervention and No Intervention by Subgroups **eTable 2.** Estimated 5-Year Risk and Risk Difference of Hospitalization for Major Cardiovascular Diseases Between the Hypothetical Intervention and No Intervention for the Main Analysis and Robustness Check **eFigure 1.** Flowchart for Selection of Eligible Participants for the Stroke Cohort in the UK Biobank **eFigure 2.** Flowchart for Selection of Eligible Participants for the Myocardial Infarction Cohort in the UK Biobank **eFigure 3.** Flowchart for Selection of Eligible Participants for the Heart Failure Cohort in the UK Biobank **eFigure 4.** Flowchart for Selection of Eligible Participants for the Arrhythmia Cohort in the UK Biobank
eReferences

Supplement 2.
Data Sharing Statement
