# Supplementary material for: A Hypothetical PM2.5 Intervention for the Risk of Hospitalization for Cardiovascular Diseases
Source: JAMA Netw Open. 2025 Oct 28;8(10):e2539862. doi: 10.1001/jamanetworkopen.2025.39862 (PMC12569718; doi:10.1001/jamanetworkopen.2025.39862)
Supplement: Supplement 1. — eAppendix 1. PM2.5 Estimates eAppendix 2. Historical Residential Location eMethods. Additional Detail on Statistical Analysis eTable 1. Estimated 5-Year Risk and Risk Difference of Hospitalization for Major Cardiovascular Diseases Between the Hypothetical Intervention and No Intervention by Subgroups eTable 2. Estimated 5-Year Risk and Risk Difference of Hospitalization for Major Cardiovascular Diseases Between the Hypothetical Intervention and No Intervention for the Main Analysis and Robustness Check eFigure 1. Flowchart for Selection of Eligible Participants for the Stroke Cohort in the UK Biobank eFigure 2. Flowchart for Selection of Eligible Participants for the Myocardial Infarction Cohort in the UK Biobank eFigure 3. Flowchart for Selection of Eligible Participants for the Heart Failure Cohort in the UK Biobank eFigure 4. Flowchart for Selection of Eligible Participants for the Arrhythmia Cohort in the UK Biobank eReferences [file jamanetwopen-e2539862-s001.pdf]

Supplementary Online Content

Lin C, Chu L, Liu R, et al. A hypothetical $PM_{2.5}$ intervention for the risk of hospitalization for cardiovascular diseases. *JAMA Netw Open.* 2025;8(10):e2539862.
doi:10.1001/jamanetworkopen.2025.39862

eAppendix 1. $PM_{2.5}$ Estimates

eAppendix 2. Historical Residential Location

eMethods. Additional Detail on Statistical Analysis

eTable 1. Estimated 5-Year Risk and Risk Difference of Hospitalization for Major Cardiovascular Diseases Between the Hypothetical Intervention and No Intervention by Subgroups

eTable 2. Estimated 5-Year Risk and Risk Difference of Hospitalization for Major Cardiovascular Diseases Between the Hypothetical Intervention and No Intervention for the Main Analysis and Robustness Check

eFigure 1. Flowchart for Selection of Eligible Participants for the Stroke Cohort in the UK Biobank

eFigure 2. Flowchart for Selection of Eligible Participants for the Myocardial Infarction Cohort in the UK Biobank

eFigure 3. Flowchart for Selection of Eligible Participants for the Heart Failure Cohort in the UK Biobank

eFigure 4. Flowchart for Selection of Eligible Participants for the Arrhythmia Cohort in the UK Biobank

eReferences

This supplementary material has been provided by the authors to give readers additional information about their work.

© 2025 Lin C et al. *JAMA Network Open*.

eAppendix 1. $PM_{2.5}$ Estimates

We developed a two-stage model to predict gapless $PM_{2.5}$ concentrations across the United Kingdom.[1] The first stage utilized co-located PM_{10} measurements to augment $PM_{2.5}$ observations, maximizing the spatiotemporal representativeness of the training data. In the second stage, we employed a light gradient boosting machine (LightGBM)[2] that combined original and augmented $PM_{2.5}$ values to model the nonlinear and high-order relationships between $PM_{2.5}$ concentrations and multi-source geospatial datasets. These datasets included meteorological factors[3-6], aerosol reanalysis[7], emission inventory[8,9], land cover data[10], road network[11], terrain data[12,13] and anthropogenic activities[14-16] as key $PM_{2.5}$ pollution drivers and spatial proxies. We optimized the weight of augmented $PM_{2.5}$ samples through iterative testing to balance data quantity and quality. The model incorporated a 10-year training period (2010-2019), extending beyond previous studies[17-19], to better capture historical trends and enable back-extrapolation of $PM_{2.5}$ concentrations before 2009. The model demonstrated strong performance, achieving grid-based cross-validation coefficients of determination (R^2) values of 0.65–0.86 for 2010-2019, and robust back-extrapolation accuracy (R^2 = 0.51–0.66) from 2006 to 2009 at the monthly level. Model interpretation through feature importance and Shapley additive explanation (SHAP) analyses confirmed alignment with established domain knowledge.

© 2025 Lin C et al. *JAMA Network Open*.

eAppendix 2. Historical Residential Location

In 2017, the UK Biobank Team compiled the participants' residential location change history based on a variety of sources, including addresses from General Practitioner (GP) systems - the most current addresses reported by participants during visits to UK Biobank assessment centers, and address changes that participants directly reported to the UK Biobank.[20] Following this, the detailed addresses were rounded by the UK Biobank Team to a 1×1 km^2 resolution. Additionally, this study enriched the location history data by incorporating the information of 'Length of time at current address' (data-field 669) gathered during the assessment center visits.

© 2025 Lin C et al. *JAMA Network Open*.

eMethods. Additional Detail on Statistical Analysis

Longitudinal targeted maximum likelihood estimation (LTMLE) is a doubly robust method comprising the process of fitting regression for the treatment mechanism and censoring mechanism using propensity score weighting and the outcome mechanism using outcome regression, which will give a consistent result if the model specification for either the treatment and censoring model (i.e., propensity score model) or the outcome model is consistent. Given that the parametric models are susceptible to model misspecification bias, particularly in high-dimensional settings,[21] we used a state-of-the-art automated machine learning framework –Autogluon[22]—for both the treatment and censoring model and the outcome model with base models of CatBoost boosted trees[23], XGBoost boosted trees[24], LightGBM boosted trees[25], generalized linear model, ridge regression model, and the weighted ensemble of the aforementioned base models. AutoGluon handles missing values by passing them to each model, which processes them using its own internal logic, thereby enhancing model diversity and improving overall ensemble performance. We used logarithmic loss and root mean square error as the evaluation metrics for the best model in classification and regression task, respectively.

We applied a robust variance estimation approach to derive the confidence interval (CI) based on the 2.5th and 97.5th percentiles of a non-parametric bootstrap distribution generated from 1 000 bootstrap samples, which mitigates the issue of anti-conservative confidence interval coverage under near positivity violation.[26]

For the estimation of the treatment and censoring model, as well as the outcome model, we adjusted for the time-invariant covariates and the full history of time-varying covariates. The time-invariant covariates included age at baseline, sex, educational attainment, average household income before tax, smoking status, and the pre-baseline variables of annual mean temperature, annual population density, annual GDHI per head index, and annual ambient $PM_{2.5}$ from year 2006 to 2014. The time-varying covariates included annual values of mean temperature, population density, GDHI per head index from the baseline to the most recent values, as well as the history of ambient $PM_{2.5}$ exposure before the current time point. The variables of annual population density and annual GDHI per head index were log-transformed when fitting the model.

© 2025 Lin C et al. *JAMA Network Open*.

eTable 1. Estimated 5-Year Risk and Risk Difference of Hospitalization for Major Cardiovascular Diseases Between the Hypothetical Intervention and No Intervention by Subgroups

Subgroup	No intervention 5-year risk (95% CI), ‰	≤ 9 μg/m³ 5-year risk (95% CI), ‰	Risk difference (95% CI), ‰
**Stroke**			
Female	9.72 (9.25, 10.17)	2.49 (2.06, 4.16)	-7.24 (-7.86, -5.51)
Male	15.43 (14.75, 16.09)	6.30 (3.62, 15.23)	-9.13 (-11.93, -0.12)
Baseline age < 68 years	7.80 (7.33, 8.23)	2.54 (2.03, 3.36)	-5.26 (-5.91, -4.34)
Baseline age ≥ 68 years	16.76 (16.14, 17.38)	8.37 (3.37, 26.56)	-8.38 (-13.40, 9.67)
**Myocardial infarction**			
Female	6.91 (6.50, 7.30)	1.61 (1.18, 2.50)	-5.30 (-5.85, -4.39)
Male	17.06 (16.35, 17.70)	3.70 (2.42, 10.95)	-13.36 (-14.79, -6.01)
Baseline age < 68 years	9.16 (8.69, 9.63)	2.76 (2.42, 3.18)	-6.39 (-6.96, -5.75)
Baseline age ≥ 68 years	13.75 (13.19, 14.36)	2.82 (2.39, 7.74)	-10.93 (-11.63, -6.02)
**Heart failure**			
Female	4.54 (4.22, 4.86)	4.81 (1.59, 11.24)	0.27 (-3.01, 6.67)
Male	8.83 (8.36, 9.33)	2.77 (2.37, 3.99)	-6.05 (-6.62, -4.76)
Baseline age < 68 years	3.64 (3.36, 3.94)	3.17 (1.28, 7.11)	-0.47 (-2.38, 3.42)
Baseline age ≥ 68 years	9.29 (8.85, 9.76)	2.48 (2.07, 3.23)	-6.81 (-7.37, -5.98)
**Arrhythmia**			
Female	14.38 (13.82, 14.99)	7.68 (3.85, 14.97)	-6.71 (-10.52, 0.69)
Male	24.28 (23.47, 25.09)	12.25 (5.50, 26.74)	-12.03 (-18.77, 2.21)
Baseline age < 68 years	13.49 (12.87, 14.06)	18.56 (4.90, 40.75)	5.07 (-8.48, 27.47)
Baseline age ≥ 68 years	24.17 (23.41, 24.94)	7.39 (4.35, 14.66)	-16.78 (-19.71, -9.49)

Intervention of reducing $PM_{2.5}$ by 5% if it is above the threshold of 9 μg/m³ among eligible participants in the UK Biobank, 2015-2019. (numeric results for Figure 2).

© 2025 Lin C et al. *JAMA Network Open*.

eTable 2. Estimated 5-Year Risk and Risk Difference of Hospitalization for Major Cardiovascular Diseases Between the Hypothetical Intervention and No Intervention for the Main Analysis and Robustness Check

Analysis	No intervention 5-year risk (95% CI), ‰	≤ 9 μg/m³ 5-year risk (95% CI), ‰	Risk difference (95% CI), ‰
**Stroke**			
Main analysis	12.32 (11.92, 12.72)	10.06 (3.37, 32.97)	-2.26 (-8.97 to 20.64)
Further adjustment for the modeled annual exposure to nitrogen dioxide and nitrogen oxides for the year 2010	12.32 (11.92, 12.72)	8.93 (3.15, 30.71)	-3.39 (-9.25 to 18.47)
Further adjustment for family history of CVD	12.32 (11.92, 12.72)	10.06 (3.05, 32.86)	-2.26 (-9.30 to 20.49)
Further adjustment for body mass index	12.32 (11.92, 12.72)	8.30 (3.12, 27.37)	-4.02 (-9.26 to 14.91)
**Myocardial infarction**			
Main analysis	11.47 (11.08, 11.84)	2.83 (2.35, 5.03)	-8.64 (-9.16, -6.38)
Further adjustment for the modeled annual exposure to nitrogen dioxide and nitrogen oxides for the year 2010	11.47 (11.08, 11.84)	2.88 (2.31, 6.02)	-8.59 (-9.21, -5.39)
Further adjustment for family history of CVD	11.47 (11.08, 11.84)	3.22 (2.62, 5.08)	-8.25 (-8.92, -6.33)
Further adjustment for body mass index	11.47 (11.08, 11.84)	2.89 (2.34, 4.96)	-8.58 (-9.17, -6.37)
**Heart failure**			
Main analysis	6.50 (6.23, 6.76)	3.29 (2.40, 5.20)	-3.20 (-4.16, -1.25)
Further adjustment for the modeled annual exposure to nitrogen dioxide and nitrogen oxides for the year 2010	6.50 (6.23, 6.76)	3.86 (2.64, 6.19)	-2.64 (-3.91, -0.29)
Further adjustment for family history of CVD	6.50 (6.23, 6.76)	3.29 (2.43, 4.84)	-3.21 (-4.09, -1.68)
Further adjustment for body mass index	6.50 (6.23, 6.76)	3.25 (2.45, 4.90)	-3.25 (-4.09, -1.52)
**Arrhythmia**			
Main analysis	18.86 (18.36, 19.35)	14.70 (6.14, 31.74)	-4.16 (-12.70, 12.93)
Further adjustment for the modeled annual exposure to nitrogen dioxide and nitrogen oxides for the year 2010	18.86 (18.36, 19.35)	15.14 (6.53, 31.54)	-3.72 (-12.31, 12.63)

© 2025 Lin C et al. *JAMA Network Open*.

Analysis	No intervention 5-year risk (95% CI), ‰	≤ 9 μg/m³ 5-year risk (95% CI), ‰	Risk difference (95% CI), ‰
Further adjustment for family history of CVD	18.86 (18.36, 19.35)	14.22 (6.50, 31.09)	-4.64 (-12.32, 12.24)
Further adjustment for body mass index	18.86 (18.36, 19.35)	13.77 (6.54, 29.36)	-5.09 (-12.29, 10.63)

Intervention of reducing $PM_{2.5}$ by 5% if it is above the threshold of 9 μg/m³ among eligible participants in the UK Biobank, 2015-2019. (numeric results for Figure 3).

© 2025 Lin C et al. *JAMA Network Open*.

eFigure 1. Flowchart for Selection of Eligible Participants for the Stroke Cohort in the UK Biobank

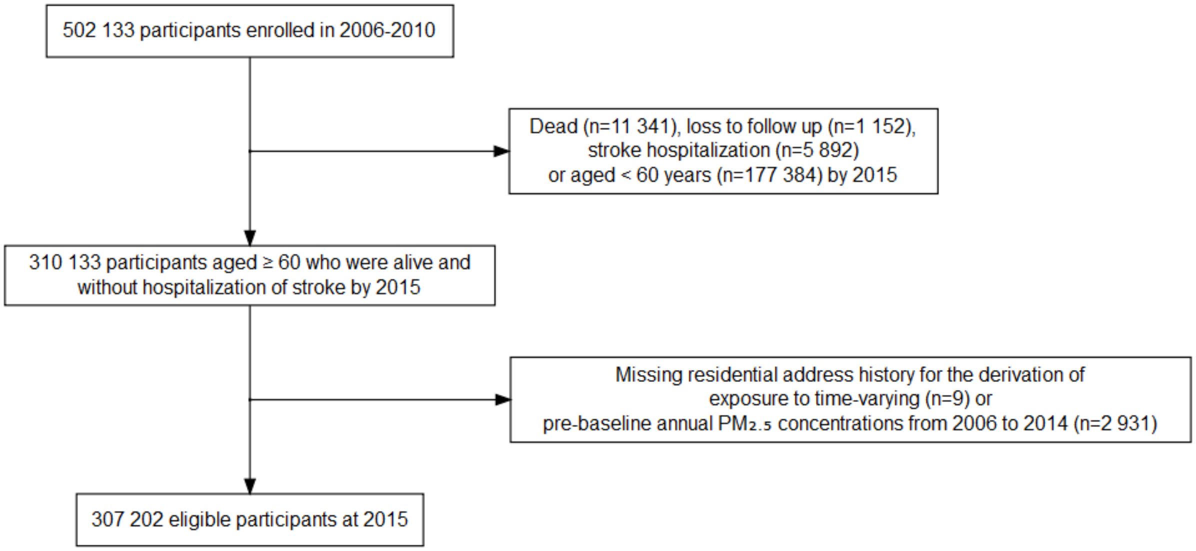

© 2025 Lin C et al. *JAMA Network Open*.

eFigure 2. Flowchart for Selection of Eligible Participants for the Myocardial Infarction Cohort in the UK Biobank

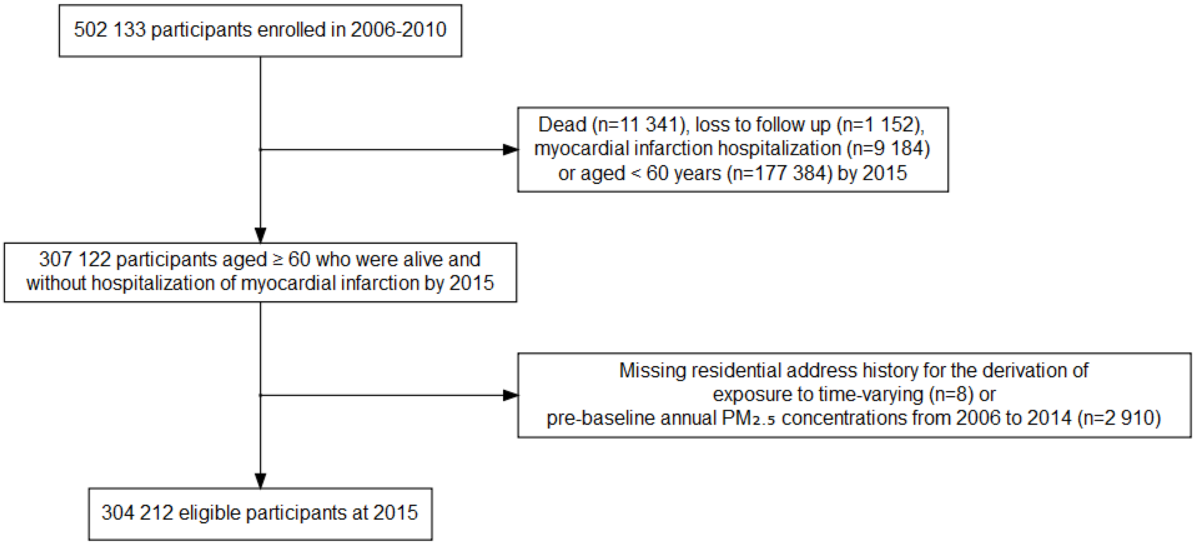

© 2025 Lin C et al. *JAMA Network Open*.

eFigure 3. Flowchart for Selection of Eligible Participants for the Heart Failure Cohort in the UK Biobank

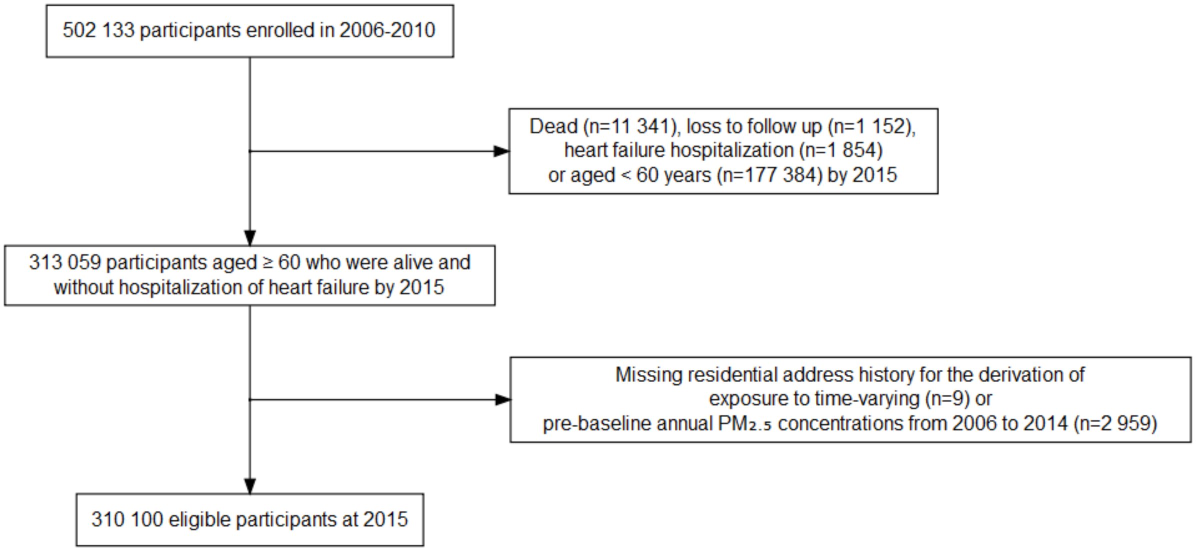

© 2025 Lin C et al. *JAMA Network Open*.

eFigure 4. Flowchart for Selection of Eligible Participants for the Arrhythmia Cohort in the UK Biobank

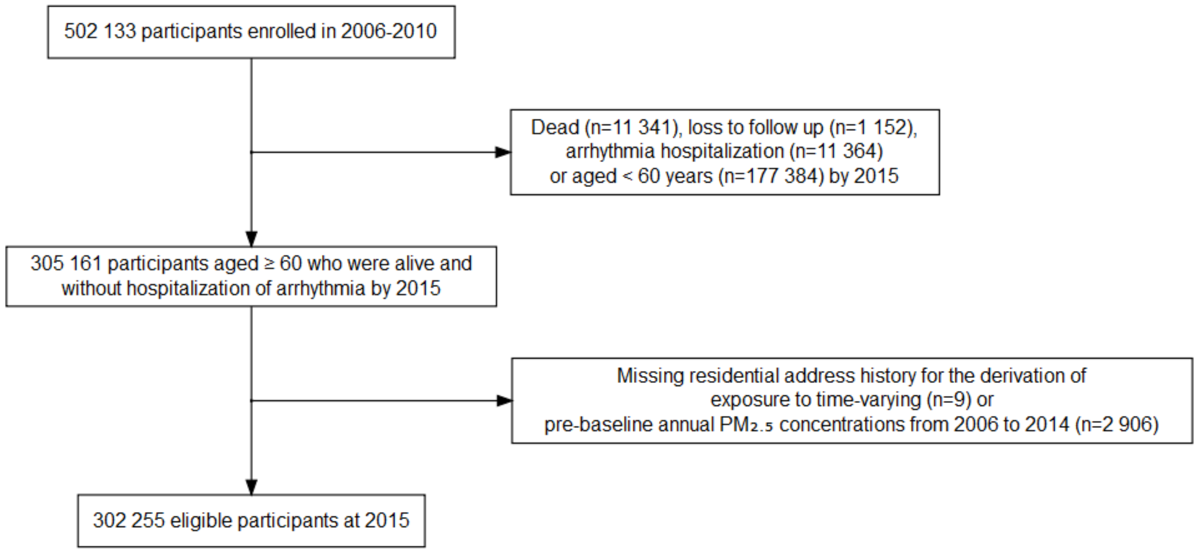

© 2025 Lin C et al. *JAMA Network Open*.

eReferences

1. Liu R, Ma Z, Gasparrini A, de la Cruz A, Bi J, Chen K. Integrating Augmented In Situ Measurements and a Spatiotemporal Machine Learning Model To Back Extrapolate Historical Particulate Matter Pollution over the United Kingdom: 1980–2019. *Environmental Science & Technology*. 2023/12/26 2023;57(51):21605-21615. doi:10.1021/acs.est.3c05424
2. Ke G, Meng Q, Finley T, et al. Lightgbm: A highly efficient gradient boosting decision tree. In: Guyon I, Luxburg UV, Bengio S, et al, eds. *Advances in neural information processing systems*. Curran Associates, Inc.; 2017.
3. Hersbach H, Bell B, Berrisford P, et al. The ERA5 global reanalysis. 2020;146(730):1999-2049. doi:https://doi.org/10.1002/qj.3803
4. Muñoz-Sabater J, Dutra E, Agustí-Panareda A, et al. ERA5-Land: a state-of-the-art global reanalysis dataset for land applications. *Earth Syst Sci Data*. 2021;13(9):4349-4383. doi:10.5194/essd-13-4349-2021
5. Perry M, Hollis D. The generation of monthly gridded datasets for a range of climatic variables over the UK. *International Journal of Climatology*. 2005;25(8):1041-1054. doi:https://doi.org/10.1002/joc.1161
6. Hollis D, McCarthy M, Kendon M, Legg T, Simpson I. HadUK-Grid—A new UK dataset of gridded climate observations. *Geoscience Data Journal*. 2019;6(2):151-159. doi:https://doi.org/10.1002/gdj3.78
7. Gelaro R, McCarty W, Suárez MJ, et al. The modern-era retrospective analysis for research and applications, version 2 (MERRA-2). *Journal of climate*. 2017;30(14):5419-5454. doi:https://doi.org/10.1175/JCLI-D-16-0758.1
8. Hoesly RM, Smith SJ, Feng L, et al. Historical (1750–2014) anthropogenic emissions of reactive gases and aerosols from the Community Emissions Data System (CEDS). *Geoscientific Model Development*. 2018;11(1):369-408. doi:https://doi.org/10.5194/gmd-11-369-2018
9. Feng L, Smith SJ, Braun C, et al. The generation of gridded emissions data for CMIP6. *Geoscientific Model Development*. 2020;13(2):461-482. doi:https://doi.org/10.5194/gmd-13-461-2020
10. Copernicus Climate Data Store. Data from: Land cover classification gridded maps from 1992 to present derived from satellite observations. 2019. doi:10.24381/cds.006f2c9a
11. Haklay M, Weber P. Openstreetmap: User-generated street maps. *IEEE Pervasive computing*. 2008;7(4):12-18. doi:https://doi.org/10.1109/MPRV.2008.80
12. NASA JPL. NASADEM Merged DEM Global 1 arc second V001. doi:https://doi.org/10.5067/MEaSUREs/NASADEM/NASADEM_HGT.001
13. NASA JPL. NASADEM Slope and Curvature Global 1 arc second V001. doi:https://doi.org/10.5067/MEaSUREs/NASADEM/NASADEM_SC.001 Accessed 2022-04-09. https://lpdaac.usgs.gov/products/nasadem_scv001/
14. Schiavina M, Freire S, MacManus K. GHS-POP R2022A - GHS population grid multitemporal (1975-2030) - OBSOLETE RELEASE. doi:https://doi.org/10.2905/D6D86A90-4351-4508-99C1-CB074B022C4A Accessed 2022-07-14. https://data.jrc.ec.europa.eu/dataset/d6d86a90-4351-4508-99c1-cb074b022c4a
15. Schiavina M, Melchiorri M, Pesaresi M. GHS-SMOD R2022A - GHS settlement layers, application of the Degree of Urbanisation methodology (stage I) to GHS-POP R2022A and GHS-BUILT-S R2022A, multitemporal (1975-2030) - OBSOLETE RELEASE. doi:https://doi.org/10.2905/4606D58A-DC08-463C-86A9-D49EF461C47F Accessed 2022-07-14. https://data.jrc.ec.europa.eu/dataset/4606d58a-dc08-463c-86a9-d49ef461c47f
16. Li X, Zhou Y, Zhao M, Zhao X. Harmonization of DMSP and VIIRS nighttime light data from 1992-2021 at the global scale. 2023-09-27. doi:https://doi.org/10.6084/m9.figshare.9828827.v8 Accessed 2024-05-28. https://figshare.com/articles/dataset/Harmonization_of_DMSP_and_VIIRS_nighttime_light_data_from_1992-2018_at_the_global_scale/9828827
17. Liu M, Bi J, Ma Z. Visibility-Based PM2.5 Concentrations in China: 1957-1964 and 1973-2014. *Environmental Science & Technology*. 2017/11/21 2017;51(22):13161-13169. doi:https://doi.org/10.1021/acs.est.7b03468
18. Araki S, Shima M, Yamamoto K. Estimating historical PM2.5 exposures for three decades (1987–2016) in Japan using measurements of associated air pollutants and land use regression. *Environmental Pollution*. 2020/08/01/ 2020;263:114476. doi:https://doi.org/10.1016/j.envpol.2020.114476

© 2025 Lin C et al. *JAMA Network Open*.

19. Zhong J, Zhang X, Gui K, et al. Reconstructing 6-hourly PM2.5 datasets from 1960 to 2020 in China. *Earth System Science Data*. 2022;14(7):3197-3211. doi:https://doi.org/10.5194/essd-14-3197-2022
20. UK Biobank. Address change history derivation. 2024. Accessed 01/01/2024, https://biobank.ndph.ox.ac.uk/crystal/refer.cgi?id=526
21. Zivich PN, Breskin A. Machine learning for causal inference: on the use of cross-fit estimators. *Epidemiology*. 2021;32(3)
22. Erickson N, Mueller J, Shirkov A, et al. Autogluon-tabular: robust and accurate automl for structured data. 2020;
23. Prokhorenkova L, Gusev G, Vorobev A, Dorogush AV, Gulin A. CatBoost: unbiased boosting with categorical features. *Advances in neural information processing systems*. 2018;31
24. Chen T, Guestrin C. XGBoost: a scalable tree boosting system. presented at: Proceedings of the 22nd ACM SIGKDD International Conference on Knowledge Discovery and Data Mining; 2016; San Francisco, California, USA. https://doi.org/10.1145/2939672.2939785
25. Ke G, Meng Q, Finley T, et al. Lightgbm: a highly efficient gradient boosting decision tree. *Advances in neural information processing systems*. 2017;30
26. Tran L, Petersen M, Schwab J, van der Laan MJ. Robust variance estimation and inference for causal effect estimation. 2023;11(1)Journal of Causal Inference. doi:10.1515/jci-2021-0067

© 2025 Lin C et al. *JAMA Network Open*.
