# Supplementary material for: A Hypothetical PM2.5 Intervention for the Risk of Hospitalization for Cardiovascular Diseases
Source: JAMA Netw Open. 2025 Oct 28;8(10):e2539862. doi: 10.1001/jamanetworkopen.2025.39862 (PMC12569718; doi:10.1001/jamanetworkopen.2025.39862)
Supplement: Supplement 2. — Data Sharing Statement [file jamanetwopen-e2539862-s002.pdf]

Data Sharing Statement

Lin. A Hypothetical $PM_{2.5}$ Intervention for the Risk of Hospitalization for Cardiovascular Diseases. *JAMA Netw Open*. Published October 28, 2025.
doi:10.1001/jamanetworkopen.2025.39862

Data
Data available: No

Additional Information
Explanation for why data not available: Access to the UK Biobank dataset can be applied for at https://ukbiobank.ac.uk/register-apply/.
